# SARS-CoV-2 vaccine acceptance among caregivers of children younger than five years of age: A cross-sectional survey in Toronto

**DOI:** 10.14745/ccdr.v49i04a05

**Published:** 2023-04-01

**Authors:** Pierre-Philippe Piché-Renaud, Elahe Karimi-Shahrbabak, Sarah Abu Fadaleh, Daniel Farrar, Julia Orkin, Michelle Science, Shaun Morris

**Affiliations:** 1 Division of Infectious Diseases, The Hospital for Sick Children, Toronto, ON; 2 Department of Pediatrics, Faculty of Medicine, University of Toronto, Toronto, ON; 3 Child Health Evaluative Sciences, The Hospital for Sick Children, Toronto, ON; 4 Division of Clinical Public Health and Centre for Vaccine Preventable Diseases, Dalla Lana School of Public Health, Toronto, ON

**Keywords:** SARS-CoV-2, COVID-19, caregivers, children, vaccine acceptance, vaccine hesitancy, survey

## Abstract

**Background:**

Despite severe acute respiratory syndrome coronavirus 2 (SARS-CoV-2) vaccine approval in Canada for children six months to five years old, vaccine acceptance for this age group remains low compared with other age groups. This study aimed to assess vaccine acceptance among caregivers of children younger than five years old and to identify factors associated with SARS-CoV-2 vaccine hesitancy in Toronto.

**Methods:**

A multi-language self-administered survey was sent to caregivers of children attending 660 Toronto schools and two community health centres between April 5 to July 4, 2022. Data on socio-demographic characteristics, acceptance of routine childhood and influenza vaccines and current SARS-CoV-2 vaccine status for parents and older siblings were collected.

**Results:**

A total of 253 caregivers of children younger than five years old answered the survey. Although 234 (94%) of the responding caregivers were fully vaccinated against SARS-CoV-2 and more than 90% had their children older than five years receiving one dose of the vaccine, only 148 (59%) had intentions to vaccinate their child younger than five years old.

**Conclusion:**

These findings highlight the importance of interventions to increase vaccine confidence among caregivers of children aged younger than five years old.

## Introduction

In Ontario, from January 15, 2020, to March 11, 2023, there have been 1,695 children younger than five years old hospitalized for coronavirus disease 2019 (COVID-19) and 23 deaths from COVID-19 ((1)). National data from Canada found that among children younger than 18 years old hospitalized because of COVID-19, the highest proportion of severe disease was in children aged 2–5 years old ((2)). On July 14, 2022, Health Canada first approved severe acute respiratory syndrome coronavirus 2 (SARS-CoV-2) vaccines for children six months to five years of age ((3)). However, the uptake of SARS-CoV-2 vaccines in this age group has been low in all provinces and remains below 10% in Canada, compared with 52% in the 5–11 years old age group and 83.9% in the 12–17 years old age group ((4)). Although vaccine uptake rates (which refer to the actual behaviour of getting a vaccine) are lower than reported rates of vaccine acceptance (which refer to the intent to receive a vaccine, or attitude), understanding the COVID-19 vaccine acceptance rates in caregivers of young children and their reasons for hesitancy would allow for the development of targeted interventions to increase confidence and uptake in this age group ((5,6)). This study addresses time-sensitive and urgent public health matters: 1) the assessment of vaccine acceptance (intent to receive a vaccine or to vaccinate) in caregivers of children younger than five years of age and 2) identification of factors associated with vaccine hesitancy (delay in acceptance or refusal of vaccines) in Ontario prior to the national approval of vaccines for this age group ((6)).

## Methods

A multi-language self-administered cross-sectional survey was sent to caregivers of children and/or pre-school children at 660 public and private schools affiliated with the Hospital for Sick Children COVID-19 Testing Centre and two community health centres in Toronto. The survey was distributed from April 5 to July 4, 2022—immediately before the approval of the vaccine for children six months to five years of age. Caregivers who reported caring for children younger than five years old were asked about their intention to vaccinate their child(ren) against SARS-CoV-2 upon approval of the vaccine for this age group, and their reasons for being hesitant to accept COVID-19 vaccines. Caregivers were considered vaccine-acceptant when they intended (very likely/likely) to vaccinate their children against SARS-CoV-2 in the future, and vaccine-hesitant when had little or no intention (very unlikely/unlikely/unsure) to vaccinate their children in the future. The survey collected information on socio-demographic characteristics (including age, relationship to the child, education status, country of birth and ethnicity) and current vaccine status for caregivers and older siblings of the school-aged group. The questionnaire can be found in the **Supplemental material**. Data were analyzed using χ^2^ or Fisher’s exact tests and *p*-values of <0.05 were considered to be statistically significant. Responses to the open-ended questions were coded and clustered using thematic analysis.

## Results

A total of 253 caregivers of children younger than five years old answered the survey. Although 234 (94%) of the responding caregivers had received at least two doses of COVID-19 vaccine and more than 90% had their children older than five years receiving at least one dose of the vaccine, only 148 (59%) intended to vaccinate their child(ren) younger than five years old. The level of COVID-19 vaccine acceptance differed among caregivers of different ethnic backgrounds, with lowest acceptance reported in Black (25%) and Middle Eastern (37.5%) (*p*=0.006). Other characteristics associated with differences in vaccine acceptance included caregiver’s age (*p*=0.039, lowest in those over 40 years of age), education level (*p*=0.011, lowest in university graduates) and vaccination status (*p*<0.001, lowest in unvaccinated caregivers) (**Table 1**). Caregivers reported seeking information on COVID-19 vaccines primarily from public health resources (79%), government organizations (62%), social media (58%) and family doctors or paediatricians (45%). When comparing caregivers with different ethnic backgrounds, there were significant differences in the number of those seeking information from public health resources (*p*<0.001) and family doctors or paediatricians (*p*<0.001). Compared with caregivers with White background, all other ethnic backgrounds had lower reports of seeking information from public health resources (*p*<0.001) and family doctors or paediatricians (*p*<0.001). Additionally, seeking information from family doctors or paediatricians (*p*=0.029), public health resources (*p*<0.001), government organizations (*p*<0.001), professional groups (*p*=0.012) and social media (*p*=0.001) differed among caregivers with different levels of education. Caregivers with higher levels of education had higher reports of getting information from family doctors or paediatricians (except for those less than high school), public health resources, government organizations, professional groups and social media (except for a community college diploma) than those with lower levels of education (**Table 2**).

**Table 1:** SARS-COV-2 vaccine acceptance among caregivers of children younger than five years of age

Characteristic of caregiver	All participants (N=253)		Vaccine acceptance^a^				*p-*value
			Acceptant		Hesitant		
			(N=148, 58.5%)		(N=105, 41.5%)		
	n/N	%	n/N	%	n/N	%	
**Relationship to child**							
Father	52/253	20.6	35/52	67.3	17/52	32.7	0.34
Mother	199/253	78.7	112/199	56.3	87/199	43.7	
Grandparent	2/253	0.7	1/2	50.0	1/2	50.0	
**Ethnicity^b^**							
White	105/244	43.0	68/105	64.8	37/105	35.2	0.006
East/Southeast Asian	49/244	20.1	35/49	71.4	14/49	28.6	
South Asian	28/244	11.5	16/28	57.1	12/28	42.9	
Black	24/244	9.8	6/24	25.0	18/24	75.0	
Mixed and other race category	17/244	7.0	10/17	58.8	7/17	41.2	
Latino	13/244	5.3	7/13	53.8	6/13	46.2	
Middle Eastern	8/244	3.3	3/8	37.5	5/8	62.5	
**Age group**							
Younger than 30 years	5/253	2.0	0/5	0.0	5/5	100.0	0.039
30–39 years	148/253	58.5	86/148	58.1	62/148	41.9	
40–49 years	95/253	37.5	58/95	61.1	37/95	38.9	
50 years or older	5/253	2.0	4/5	80.0	1/5	20.0	
**Education status**							
Less than high school	5/243	2.1	3/5	60.0	2/5	40.0	0.011
High school or equivalent	23/243	9.5	11/23	47.8	12/23	52.2	
Community college	42/243	17.3	17/42	40.5	25/42	59.5	
University graduate	173/243	71.2	114/173	65.9	59/173	34.1	
**Country of birth**							
Canada	127/249	51.0	80/127	63.0	47/127	37.0	0.24
Other countries	122/249	49.0	68/122	55.7	54/122	44.3	
**Country of birth income level^c^**							
Low/lower middle income	50/247	20.2	29/50	58.0	21/50	42.0	0.81
High/upper middle income	197/247	79.8	118/197	59.9	79/197	40.1	
**Has family doctor or paediatrician**							
Yes	240/251	95.6	139/240	57.9	101/240	42.1	0.766
No	11/251	4.4	7/11	63.6	4/240	36.4	
**Number of children**							
1	27/250	10.8	17/27	63.0	10/27	37.0	0.595
2	143/250	57.2	85/143	59.4	58/143	40.6	
3	58/250	23.2	35/58	60.3	23/58	39.7	
4 or more	22/250	8.8	10/22	45.5	12/22	54.5	
**Caregiver’s SARS-CoV-2 vaccination status**							
Received at least one dose	236/252	93.7	147/236	62.3	89/236	37.7	<0.001
Has not received any dose	16/252	6.4	1/16	6.3	15/16	93.8	
**Sibling’s (aged 12–18 years) SARS-CoV-2 vaccination status**							
Received at least one dose	33/46	71.7	21/33	63.6	12/33	36.4	0.007
Has not received any dose	13/46	28.3	2/13	15.4	11/13	84.6	
**Sibling’s (aged 5–11 years) SARS-CoV-2 vaccination status**							
Received at least one dose	136/195	69.7	109/136	80.1	27/136	19.9	<0.001
Has not received any dose	59/195	30.3	6/59	10.2	53/59	89.8	
**Current sources of information on SARS-CoV-2 vaccines^d^**							
Family doctor or paediatrician	113/251	45.0	71/113	62.8	42/113	37.2	0.21
Public health resources	197/251	78.5	122/197	61.9	75/197	38.1	0.038
Government organizations	156/251	62.3	96/156	61.5	60/156	38.5	0.21
Professional groups^e^	56/251	22.3	37/56	66.1	19/56	33.9	0.19
Social media^f^	145/251	57.8	88/145	60.7	57/145	39.3	0.41
Social network^g^	81/251	32.3	49/81	60.5	32/81	39.5	0.66
Other sources^h^	17/251	68.0	3/17	17.6	14/17	82.4	<0.001

Abbreviation: SARS-COV-2, severe acute respiratory syndrome coronavirus 2

^a^ Caregivers were considered vaccine-acceptant when they intended (very likely/likely) to vaccinate their children against SARS-CoV-2 in the future, and vaccine-hesitant when had little or no intention (very unlikely/unlikely/unsure) to vaccinate their children in the future

^b^ Caregivers were able to choose multiple responses for their ethnicity, the Indigenous ethnic group was classified as “mixed and other” due to small group size

^c^ Caregivers’ country of birth income level was defined based on the World Bank classification 2021 ((7))

^d^ Caregivers were able to choose multiple answers for the current source of information

^e^ Professional groups such as Canadian Medical Association, Canadian Nurses Association, etc.

^f^ Social media includes Twitter, Facebook, WhatsApp, WeChat, radio/television/newspapers or news websites

^g^ Social networks such as family, friends, neighbours or co-workers. Percentages were calculated based on the cases with complete data

^h^ Other sources reported in open-ended questions include scientific articles, workplaces, schools, statistics on government websites, World Health Organization and Centers for Disease Control and Prevention reports, and a friend (a virologist)

**Table 2:** Current sources of information on SARS-CoV-2 vaccines among caregivers of children younger than five years of age

Characteristic of caregiver	All participants (N=251)^a^		Current sources of information on SARS-CoV-2 vaccines													
			Family doctor or paediatrician (N=113, 45.0%)		Public health resources (N=197, 78.5%)		Government organizations (N=156, 62.2%)		Professional groups (N=56, 22.3%)		Social media (N=145, 57.8%)		Social network (N=81, 32.3%)		Other sources (N=17, 6.8%)	
	n/N	%	n/N	%	n/N	%	n/N	%	n/N	%	n/N	%	n/N	%	n/N	%
**Ethnicity** **(*p*-value)**	**N/A**		**<0.001**		**<0.001**		**0.658**		**0.54**		**0.059**		**0.902**		**0.233**	
White	105/242	43.4	64/105	61.0	97/105	92.4	71/105	67.6	30/105	28.6	64/105	61.0	31/105	29.5	9/105	8.6
East/Southeast Asian	48/242	19.8	12/48	25.0	39/48	81.3	30/48	62.5	8/48	16.7	35/48	72.9	17/48	35.4	1/48	2.1
South Asian	28/242	11.6	10/28	35.7	20/28	71.4	15/28	53.6	4/28	14.3	13/28	46.4	8/28	28.6	0/28	0.0
Black	24/242	9.9	11/24	45.8	15/24	62.5	16/24	66.7	4/24	16.7	13/24	54.2	6/24	25.0	2/24	8.3
Mixed and other race category	16/242	6.6	4/16	25.0	9/16	56.3	9/16	56.3	4/16	25.0	8/16	50.0	7/16	43.8	2/16	12.5
Latino	13/242	5.4	6/13	46.2	8/13	61.5	7/13	53.8	2/13	15.4	5/13	38.5	5/13	38.5	0/13	0.0
Middle Eastern	8/242	3.3	3/8	37.5	5/8	62.5	4/8	50.0	1/8	12.5	2/8	25.0	2/8	25.0	1/8	12.5
**Age group** **(*p*-value)**	**N/A**		**0.597**		**0.48**		**0.64**		**0.655**		**0.299**		**0.41**		**0.42**	
Younger than 30 years	4/251	1.6	1/4	25.0	4/4	100.0	3/4	75.0	1/4	25.0	3/4	75.0	2/4	50.0	0/4	0.0
30–39 years	148/251	59.0	64/148	43.2	118/148	79.7	87/148	58.8	31/148	20.9	84/148	56.8	52/148	35.1	11/148	7.4
40–49 years	94/251	37.5	46/94	48.9	70/94	74.5	62/94	66.0	24/94	25.5	53/94	56.4	25/94	26.6	5/94	5.3
50 years and older	5/251	2.0	2/5	40.0	5/5	100.0	4/5	80.0	0/5	0.0	5/5	100.0	2/5	40.0	1/5	20.0
**Education status** **(*p*-value)**	**N/A**		**0.029**		**<0.001**		**<0.001**		**0.012**		**0.001**		**0.323**		**0.445**	
Less than high school	5/241	2.1	3/5	60.0	2/5	40.0	2/5	40.0	0/5	0.0	0/5	0.0	0/5	0.0	1/5	20.0
High school or equivalent	122/241	50.6	10/22	45.5	13/22	59.1	7/22	31.8	1/22	4.5	7/22	31.8	5/22	22.7	1/22	4.5
Community college	42/241	17.4	11/42	26.2	25/42	59.5	23/42	54.8	6/42	14.3	27/42	64.3	15/42	35.7	3/42	7.1
University graduate	172/241	71.4	87/172	50.6	150/172	87.2	120/172	69.8	49/172	28.5	106/172	61.6	57/172	33.1	11/172	6.4

Abbreviations: N/A, not applicable; SARS-COV-2, severe acute respiratory syndrome coronavirus 2

^a^ For the question on current sources of information, there were two missing answers

**Figure 1:**
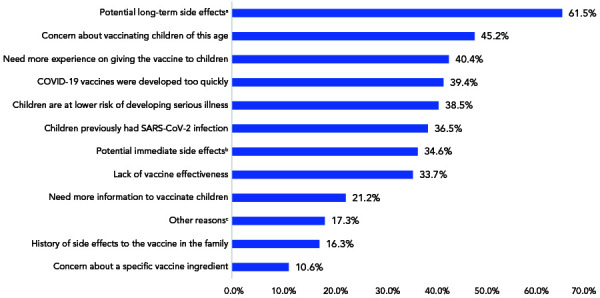
Reasons for SARS-CoV-2 vaccine hesitancy in caregivers of children younger than five years of age^a,b,c^ Abbreviations: COVID-19, coronavirus disease 2019; SARS-COV-2, severe acute respiratory syndrome coronavirus 2 ^a^ Long-term side effects reported in open-ended questions include cardiovascular (11/64 or 17.2%; e.g. myocarditis, pericarditis, bleeding and other cardiovascular effects), neurological or developmental (6/64 or 9,4%; e.g. developmental or cognitive issues, stroke and aneurism and other neurological side effects), uncertain due to lack of data, evidence for and potential unspecified new side effects (20/64 or 31.3%), infertility (4/64 or 6.3%), inflammation and autoimmune disease (3/60 or 4.7%) and other long-term side effects (6/64 or 25.0%) ^b^ Immediate side effects include cardiovascular (11/36 or 30.6%; e.g. myocarditis/pericarditis, thrombosis and other cardiovascular effects), neurological (3/36 or 8.3%; e.g. stroke, Bell’s palsy or other forms of paralysis and seizure), vaccine reactogenicity (7/36 or 19.4%; e.g. fever and pain), allergic reactions (2/36 or 5.6%); inflammation and autoimmune disease (2/36 or 5.6%) and other immediate side effects (11/36 or 31%) ^c^ Other reasons include vaccination being unnecessary (4/18 or 22.2%), lack of effectiveness (3/18 or 16.7%) and other reasons (5/18 or 27.8%; e.g. other specific concerns and other general and unspecified concerns)

Among caregivers who were vaccine hesitant (105 caregivers with one participant not providing a reason), the most common reason for vaccine hesitancy was the concern about long-term side effects (n=64/104; 62%). A third of caregivers who were hesitant to vaccinate their child(ren) reported concerns around the lack of data and evidence on COVID-19 vaccines, reported immediate side effects from vaccines and the potential for new unspecified side effects. Additional concerns included children being too young to be vaccinated (n=47/104; 45%) and wanting to wait until there is more experience with vaccinating children in this age group (n=42/104; 40%). Among caregivers with concerns about long-term side effects reported in open-ended questions, 11 (17.2%) were concerned about cardiovascular side effects, six (9.4%) about neurological or developmental side effects, four (6.3%) about infertility, three (4.7%) about inflammation and autoimmune disease and 16 (25.0%) about other long-term side effects (**Figure 1**).

## Conclusion

In this study, conducted prior to approval in Canada for COVID-19 vaccination in children between six months and five years of age, we found that caregivers’ intent to vaccinate their child younger than five years old was low. Although vaccine acceptance and uptake may not necessarily be identical concepts, interestingly, the proportion of caregivers who were found to be acceptant of COVID-19 vaccines for their child was found to be much lower than the proportion of caregivers who reported to be vaccinated or who had an older child that was already vaccinated against COVID-19 ((6)). These findings highlight that targeted interventions are needed to support caregivers with education and opportunities for enhanced discussion supporting COVID-19 vaccination decisions for their young children, especially in groups that were found to have lower vaccine acceptance. Healthcare and public health professionals play a crucial role in fostering SARS-CoV-2 vaccine confidence in parents and relaying up-to-date and accurate information on the benefits and risks of vaccination to caregivers of young children.

## Supplemental material

These documents can be accessed on the Supplemental material file.
Survey instrument
